# Electroacupuncture rewires interhemispheric homotopic connectivity to promote motor recovery after ischemic stroke

**DOI:** 10.3389/fneur.2026.1688307

**Published:** 2026-01-20

**Authors:** Baokai Dou, Xiangxin Xing, Zexi Dou, Lv Gao, Hairu Huo, Jing Meng, Yuling Liu, Feng Sui

**Affiliations:** 1 Institute of Chinese Materia Medica, China Academy of Chinese Medical Sciences, Beijing, China; 2 Department of Pharmacy, Shandong Provincial Hospital Affiliated to Shandong First Medical University, Jinan, Shandong, China; 3 Rehabilitation Center, Qilu Hospital of Shandong University, Jinan, China; 4 School of Special Education and Rehabilitation, Binzhou Medical University, Yantai, China

**Keywords:** stroke, electroacupuncture, voxel-mirrored homotopic connectivity, middle cerebral artery occlusion/reperfusion, fMRI

## Abstract

**Objective:**

To investigate electroacupuncture (EA)-induced reorganization of voxel-mirrored homotopic connectivity (VMHC) and its association with motor recovery in a rat model of ischemic stroke.

**Methods:**

Twenty-four female Sprague–Dawley rats were randomized into Sham group, stroke model group and EA group. The EA group received 1-week treatment (2/15 Hz sparse-dense wave, 2 mA, 30 min/day) at GV20. Neurological deficits were assessed using the modified neurological severity score. And resting-state functional magnetic resonance imaging was acquired pre-post-intervention for VMHC analysis. Group differences and VMHC-behavior correlations were evaluated.

**Results:**

EA significantly increased VMHC in subcortical motor regions (*p* = 0.001, alphasim correction) but decreased VMHC in somatosensory cortex versus untreated stroke. Model group showed progressive VMHC reductions in hippocampus, hypothalamus, and somatosensory cortex. Enhanced internal capsule VMHC correlated with improved Longa scores in EA rats (*r*^2^ = 0.206, *p* = 0.009).

**Conclusion:**

EA promotes motor recovery through frequency-specific bidirectional VMHC modulation. This study elucidates EA’s inter-hemispheric connectivity level therapeutic mechanism for stroke rehabilitation.

## 1 Introduction

Stroke remains a leading cause of mortality and disability worldwide, imposing a significant burden on healthcare systems and patients’ quality of life (1). Ischemic stroke, characterized by focal cerebral infarction due to vascular occlusion, frequently results in motor impairments such as hemiparesis, which affects over 85% of survivors (2, 3). These deficits were caused not only by localized neuronal death but also by disruptions in interhemispheric imbalance and large-scale functional and structural brain networks, including the sensorimotor network (SMN), default mode network (DMN), and subnetwork of cortico-subcortical brain (4–6). Post-stroke recovery involves complex neuroplastic mechanisms, such as peri-infarct reorganization, interhemispheric rebalancing, and compensatory recruitment of unaffected regions (7). However, recovery remains incomplete in many patients, highlighting the need for interventions that target neural reorganization at the local, interhemispheric, and network level.

Electroacupuncture (EA), a modernized form of traditional acupuncture that integrates electrical stimulation with needle insertion, has emerged as a promising adjunct therapy for stroke rehabilitation (8). Clinical studies demonstrated that EA enhances motor recovery by modulating cortical excitability, promoting neurogenesis, and improving functional connectivity (FC) between motor-related brain regions (9). For instance, EA at acupoints such as Yanglingquan (GB34) has been shown to rebalance interhemispheric compensation and elicit a combined effect of brain networks, thereby facilitating motor coordination in stroke patients (10, 11). Neuroimaging evidence further reveals that stroke induced static and dynamic reorganization of voxel-mirrored homotopic connectivity (VMHC), a metric quantifying the synchrony of spontaneous neural activity between geometrically mirrored brain regions (12). VMHC, as a noticeable indicator of the brain’s essential functional architecture of interhemispheric integration, has an important influence on cognition and behavior by interhemispheric communication. Decreased VMHC in motor-related areas, such as M1 and supplementary motor areas, and in higher-order networks, such as the DMN, are hallmarks of stroke-induced network dysfunction (13). Conversely, increased VMHC for post-stroke recovery was positively associated with better clinical outcomes. Although relevant studies have confirmed that acupuncture-driven VMHC alterations reflect functional recovery, the neuroimaging basis of EA-induced VMHC recovery remains poorly characterized, especially in animal models where controlled mechanisms are explored (14).

Rodent ischemic stroke models, represented by the middle cerebral artery occlusion/reperfusion (MCAO/R) model, provide a unique opportunity to investigate EA mechanisms at circuit levels, but few studies have explored their impact on VMHC (15, 16). Existing rodent fMRI studies have focused on functional connectivity within unilateral hemispheres or specific resting-state networks, neglecting interhemispheric homologous coordination. However, no systematic studies have integrated EA intervention with longitudinal VMHC analysis in rodent model to disentangle therapy-driven reorganization from spontaneous recovery (17).

The current study addresses these limitations by examining EA-induced alterations in VMHC in a rat model of ischemic stroke. We hypothesize that EA intervention will promote motor recovery by restoring homotopic connectivity in motor-related regions. We aim to map EA-driven changes in VMHC during the intervention; and to correlate these changes with behavioral outcomes. By delineating how EA reshapes interhemispheric communication, the study will clarify the neurobiological underpinnings of EA in stroke rehabilitation, offer a foundation for targeted neuromodulation therapies. And it bridges translational gaps between clinical observations and preclinical mechanisms, advancing our understanding of EA as a neuromodulatory therapy for stroke rehabilitation.

## 2 Methods and analysis

### 2.1 Animals

Twenty-four clean-grade female Sprague–Dawley rats (weight: 250–280 g) were obtained from the Laboratory Animal Research Center of Shandong Provincial Hospital Affiliated to Shandong First Medical University. This sample size (*n* = 8 per group) was determined to provide sufficient statistical power. Rats were housed under controlled environmental conditions with a temperature of 23 ± 2 °C, a 12-h light/dark cycle, and free access to standard rodent chow and water. All experimental procedures involving animals were approved by the Institutional Animal Care and Use Committee of Shandong Provincial Hospital Affiliated to Shandong First Medical University, and conducted in accordance with institutional and national guidelines for the care and use of laboratory animals. In this study, animals were euthanized by CO_2_ inhalation at the end of the experimental.

### 2.2 Middle cerebral artery occlusion/reperfusion model

**Figure 1:**
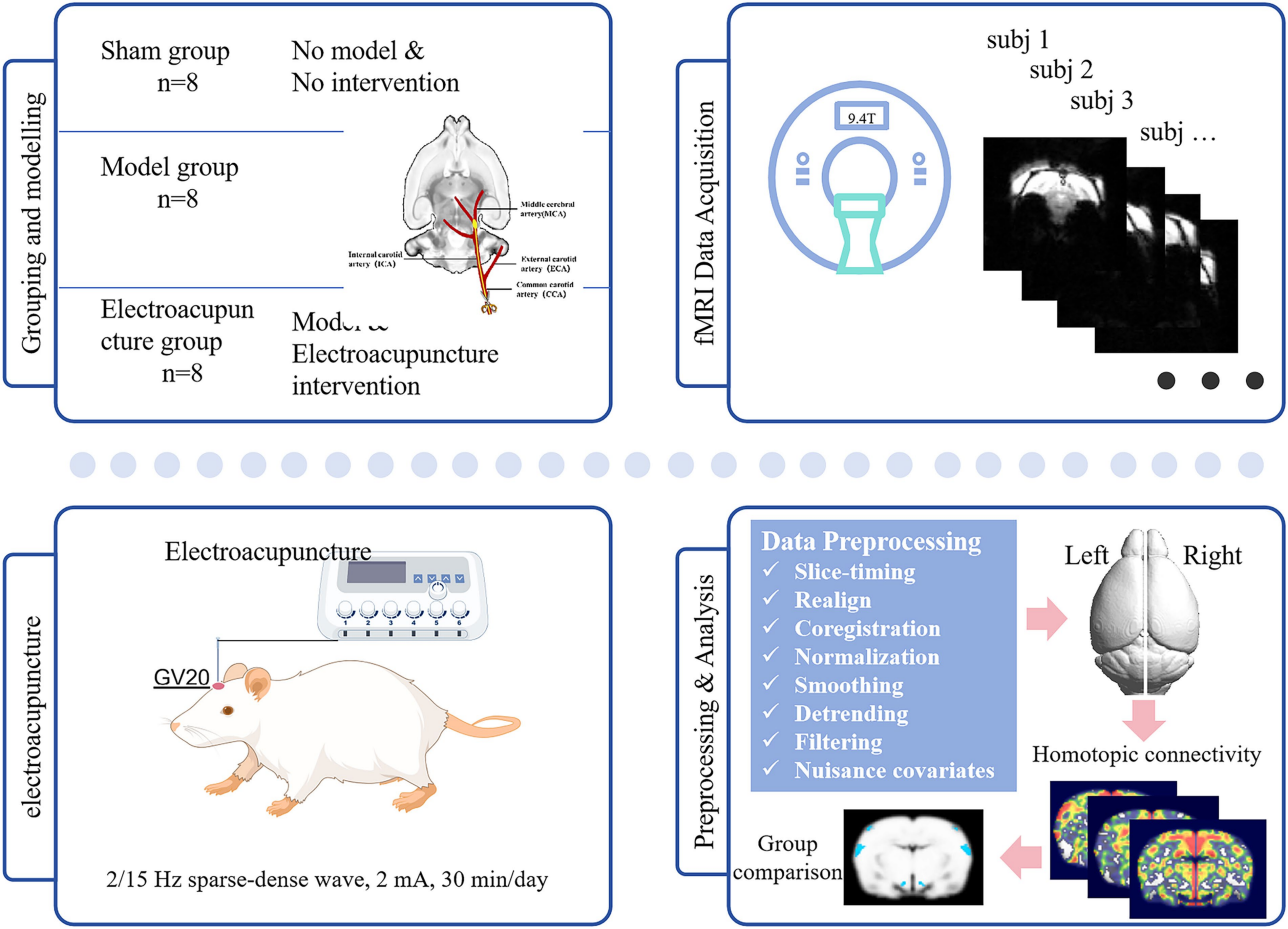
Experimental flowchart. GV 20, Governor Vessel 20. Icons in the part of Electroacupuncture were obtained from Figdraw.

Rats were randomly assigned to three groups (*n* = 8 per group): (1) Sham group, (2) Model group, and (3) EA group. Transient focal cerebral ischemia was induced using the intraluminal filament method to occlude the left middle cerebral artery for 60 min, followed by reperfusion. Sham-operated rats underwent identical surgical procedures except for filament insertion. During surgery and recovery, body temperature was maintained at 37.0 ± 0.5 °C using a heating pad and monitored with a rectal probe. Post-operatively, rats were placed in a temperature-controlled recovery chamber until fully ambulatory and subsequently housed individually. Food and water were provided in easily accessible low-profile bowls within the cage. Animals exhibiting severe distress or significant weight loss (>20% baseline body weight) meeting predefined humane endpoints were euthanized. The experimental flowchart is shown in Figure 1.

### 2.3 Electroacupuncture intervention

Animals were anesthetized by intraperitoneal injection of 2% chloral hydrate (400 mg/kg). No adverse effects such as peritonitis or pain-related behaviors were observed following administration. The depth of anesthesia was confirmed by the absence of pedal and corneal reflexes before surgery. And then the “Baihui (GV20)” acupoint was stimulated electrically at an intensity of 2 mA (producing visible twitching of the limb musculature) and a sparse-dense wave with 2/15 Hz frequency (18). Numerous preclinical studies have demonstrated that EA at GV20 can improve neurobehavioral outcomes of rodent stroke models, reduce infarct volume, and promote neurogenesis and angiogenesis (19, 20). A fine needle (0.25 mm in diameter, 13 mm in length) placed at GV20 was connected to one electrode of an SDZ-II EA stimulator, while the other electrode was attached to the tail to complete the circuit. Daily EA sessions were used for 30 min per day for 5 days, a regimen widely used and well-tolerated in rodent models of stroke (21). The core body temperature of rats undergoing anesthesia and EA treatment were maintained at 37.0 ± 0.5 °C by a heating pad. Rats in the sham group and model group underwent the same anesthesia treatment but did not receive acupuncture or EA.

### 2.4 Neurological deficit scores

The neurological deficit score was assessed using the modified neurological severity score (Mnss) at 24 h, 3 days, and 7 days after reperfusion by an observer blinded to the experimental groups. Detailed procedures have been described previously (22). Higher neurological deficit scores indicate greater neurological impairment.

### 2.5 fMRI data acquisition and preprocessing

Resting-state functional magnetic resonance imaging (rs-fMRI) data were acquired on day 11 (24 h after the final EA session) using an 9.4 T small animal MRI system (Bruker BioSpec). Rats were initially anesthetized with 5% isoflurane in oxygen for induction and subsequently maintained under continuous anesthesia with 1.5% isoflurane supplemented with dexmedetomidine (0.05 mg/kg/h, i.p.). Respiratory rate was continuously monitored throughout the scan using a pneumatic pillow sensor. Rats were positioned in a stereotaxic holder within a quadrature volume coil. Functional images were acquired using an echo-planar imaging (EPI) sequence with the following parameters: 200 volumes, matrix size = 64 × 64, field of view (FOV) = 27 × 27 mm^2^, repetition time (TR) = 2,000 ms, echo time (TE) = 8.142 ms, flip angle = 90°, slice thickness = 0.3 mm, number of slices = 60, number of averages = 1. Total scan time was approximately 6 min and 40 s per animal. Anatomical reference images were also acquired. Image quality was assessed immediately post-acquisition.

Preprocessing of rs-fMRI data was performed using SPM12 and included the following steps: slice timing correction, realignment for motion correction, coregistration, spatial normalization, spatial smoothing (Gaussian kernel FWHM = 4.12*4.12*4), detrending, and band-pass filtering (0.01–0.1 Hz). Nuisance covariates (mean signals from white matter, cerebrospinal fluid, and 24 motion parameters) were regressed out.

### 2.6 Voxel-mirrored homotopic connectivity analysis

VMHC analysis was conducted using the REST toolkit. For each preprocessed fMRI dataset, the Pearson correlation coefficient (r) was computed between the time series of each voxel and the time series of its geometrically mirrored counterpart in the contralateral hemisphere. The resulting correlation coefficients were transformed to z-scores using Fisher’s z-transformation to improve normality for statistical analysis. These individual z-VMHC maps were entered into a group-level random-effects analysis. Statistical significance was determined using a voxel-wise threshold of *p* < 0.001 combined with a cluster-level AlphaSim correction for multiple comparisons (1,000 Monte Carlo simulations, with a cluster-defining threshold of *p* < 0.05), as implemented in the REST toolkit.

### 2.7 Correlation analysis between VMHC and behavioral scores

To investigate the relationship between changes in brain connectivity and functional recovery, partial correlation analyses were performed. Specifically, the change in z-VMHC values within regions showing significant group differences was correlated with the change in Mnss between the first and third time points in the EA and model group. Statistical significance was set at *p* < 0.05.

## 3 Results

### 3.1 Group-level differences in VMHC

**Figure 2:**
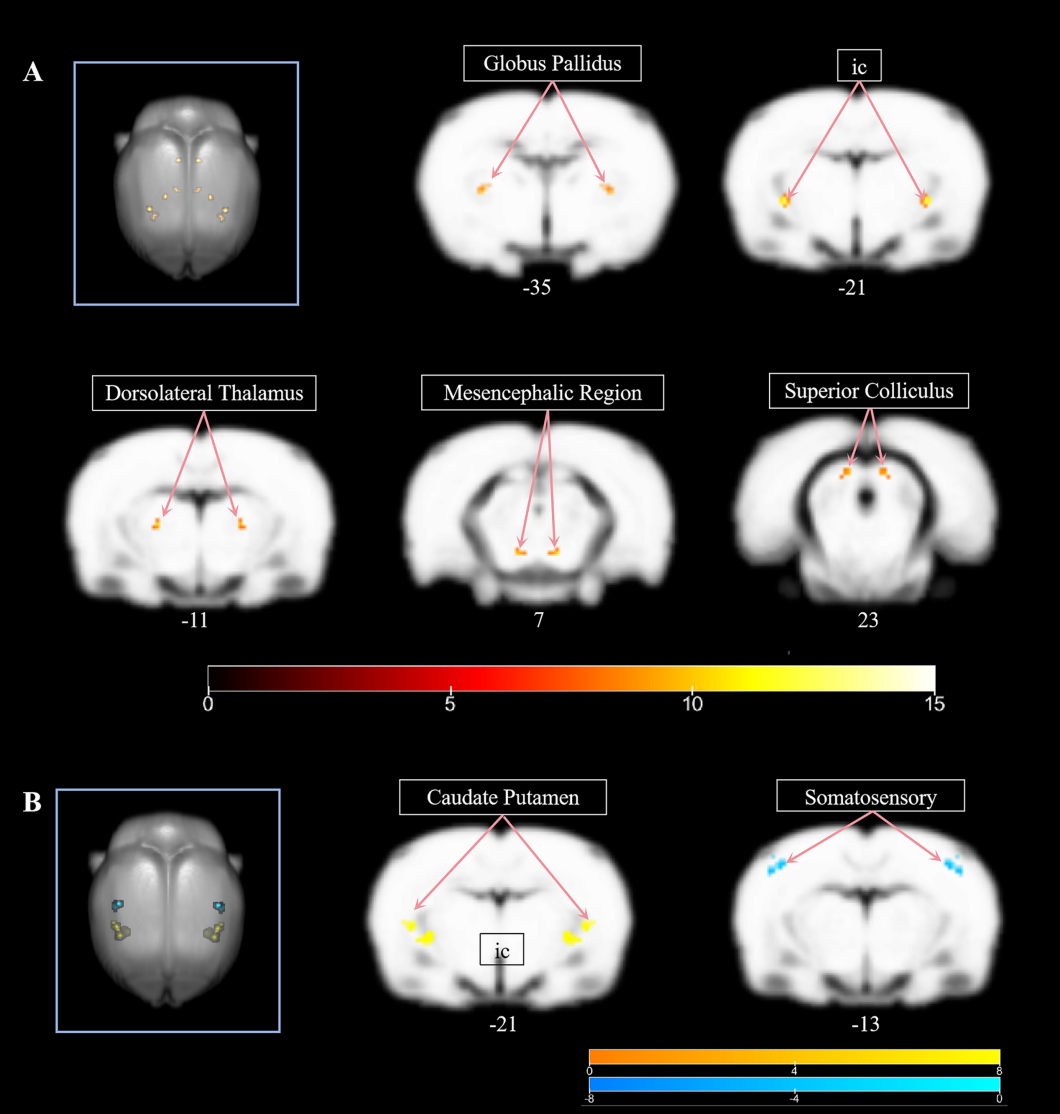
**(A)** The significant group-level differences (*F* = 8.18, *p* < 0.001) across the sham, model, and EA groups. **(B)** *Post hoc* comparisons between the EA and model groups. The warmer color bar means higher VMHC in EA group than model group, the cooler color bar means decreased VNHC in in EA group than model group.

Whole-brain voxel-wise analysis of VMHC using two-way ANOVA revealed significant group-level differences (*F* = 8.18, *p* < 0.001) across the sham, model, and EA groups. As detailed in Table 1, these differences were localized to subcortical structures critical for sensorimotor integration and basal ganglia circuitry, such as internal capsule (ic), dorsolateral thalamus, mesencephalic regiong, globus pallidus, superior colliculus. All clusters survived cluster-level correction (*p* < 0.05) and exhibited symmetric involvement across hemispheres (Figure 2A).

**Table 1:** The main effect of VMHC among the three group.

Contrast name				MNI coordinates		
	Region label	Extent	*t*-value	*x*	*y*	*z*
Positive	L_ic	15	12.7535	−40.0514	−19.6228	−21.0313
	R_ic	15	12.7535	40.2885	−19.7507	−21.0315
	L_Thalamus_Dorsolateral	7	10.9052	−23.555	−9.365	−11.0149
	R_Thalamus_Dorsolateral	7	10.9052	23.825	−9.4404	−11.0151
	L_Mesencephalic_Region	7	10.4508	−11.2278	−30.0133	6.9522
	R_Mesencephalic_Region	7	10.4508	11.4322	−30.0494	6.9521
	L_Globus_Pallidus	12	10.0876	−35.9215	−13.427	−35.0214
	R_Globus_Pallidus	12	10.0876	36.1784	−13.5419	−35.0216
	L_Superior_Colliculus	7	10.0314	−9.1022	11.1579	23.0178
	R_Superior_Colliculus	7	10.0314	9.4378	11.1283	23.0177

### 3.2 EA-induced modulation of homotopic connectivity

*Post hoc* comparisons between the EA and model groups demonstrated significant neuromodulatory effects of electroacupuncture (Table 2). Increased VMHC in ic, caudate putamen, decreased VNHC in the somatosensory cortex. This pattern indicates EA selectively enhanced subcortical interhemispheric synchrony while reducing compensatory cortical overactivation (Figure 2B).

**Table 2:** The *post hoc* analysis of VMHC of EA vs. model after intervention.

Contrast name				MNI coordinates		
	Region label	Extent	*t*-value	*x*	*y*	*z*
Positive	L_ic	120	6.8235	−40.0514	−19.6228	−21.0313
	L_Caudate_Putamen	120	4.1526	−52.3851	−3.1263	−19.005
	R_ic	120	6.8235	40.2885	−19.7507	−21.0315
	R_Caudate_Putamen	120	4.1526	52.6748	−3.2936	−19.0053
Negative	L_Cortex_Somatosensory	41	−5.2773	−48.2224	23.6375	−12.9624
	R_Cortex_Somatosensory	41	−5.2773	48.5974	23.4833	−12.9626

### 3.3 Progressive network degeneration in untreated stroke

Longitudinal analysis of the model group revealed significant VMHC reductions between pre-and post-intervention timepoints (Table 3). Decreased VMHC in posterodorsal hippocampus, somatosensory cortex and medial hypothalamus.

**Table 3:** The *post hoc* analysis of VMHC of model group between time 2 and time 1.

Contrast name				MNI coordinates		
	Region label	Extent	*t*-value	*x*	*y*	*z*
Negative	R_Hippocampus_Postero_Dorsal	193	−6.8288	−0.83923	25.6125	−6.9592
	L_Hippocampus_Postero_Dorsal	193	−4.9947	−13.2255	9.1458	−2.9854
	L_Cortex_Somatosensory	131	−6.27	−64.732	5.1397	−22.9919
	R_Cortex_Somatosensory	131	−6.27	65.0479	4.933	−22.9922
	L_Hypothalamus_Medial	44	−5.5739	−11.2475	−42.335	−17.0675
	R_Hypothalamus_Medial	44	−5.5739	11.4125	−42.3711	−17.0675

**Figure 3:**
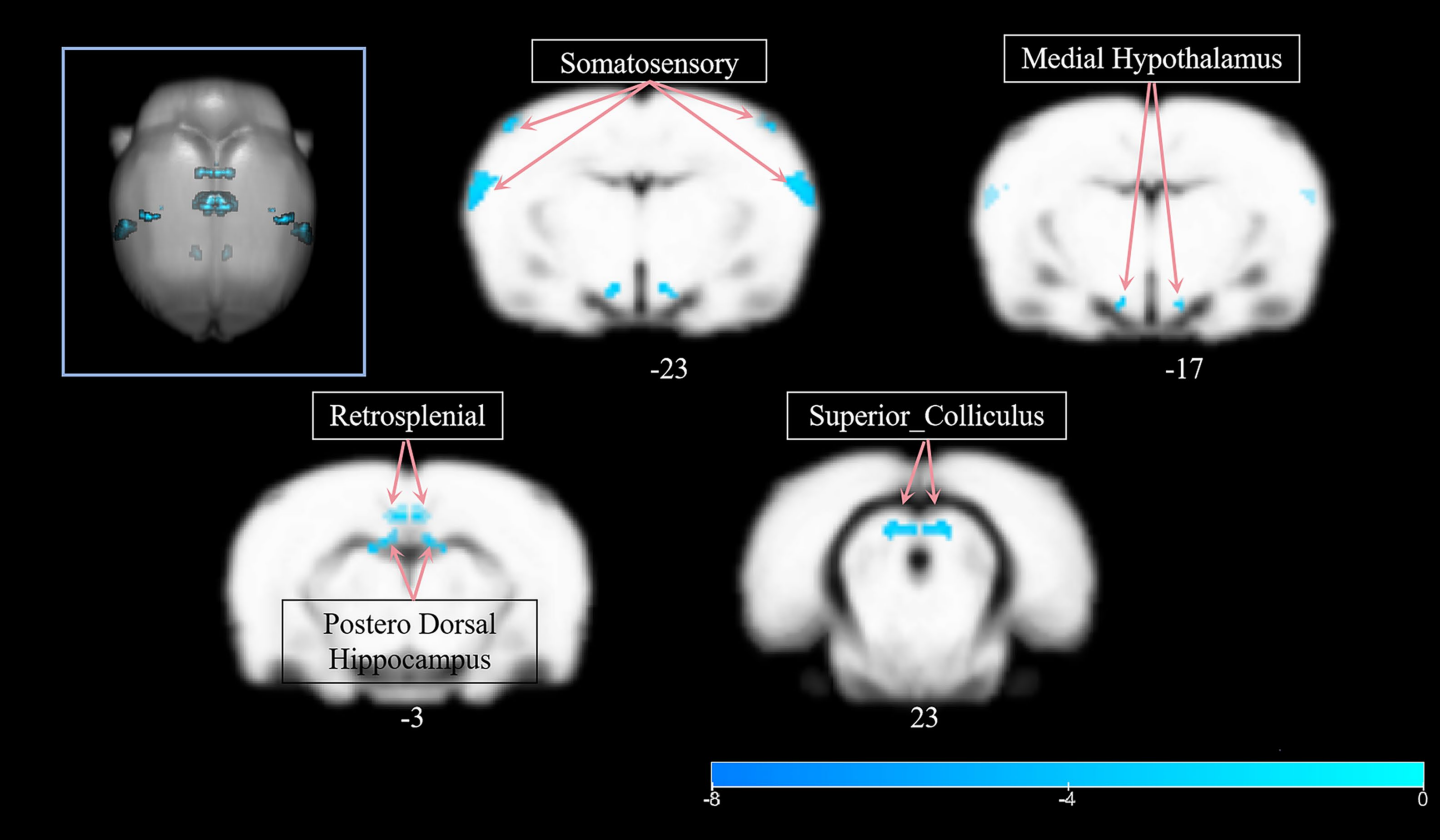
Longitudinal analysis of the model group revealed significant VMHC reductions between pre- and post-intervention timepoints.

These results demonstrate progressive decoupling of interhemispheric networks in untreated stroke, particularly affecting memory-related and sensorimotor regions (Figure 3).

### 3.4 Relationships between VMHC and behavior scores

**Figure 4:**
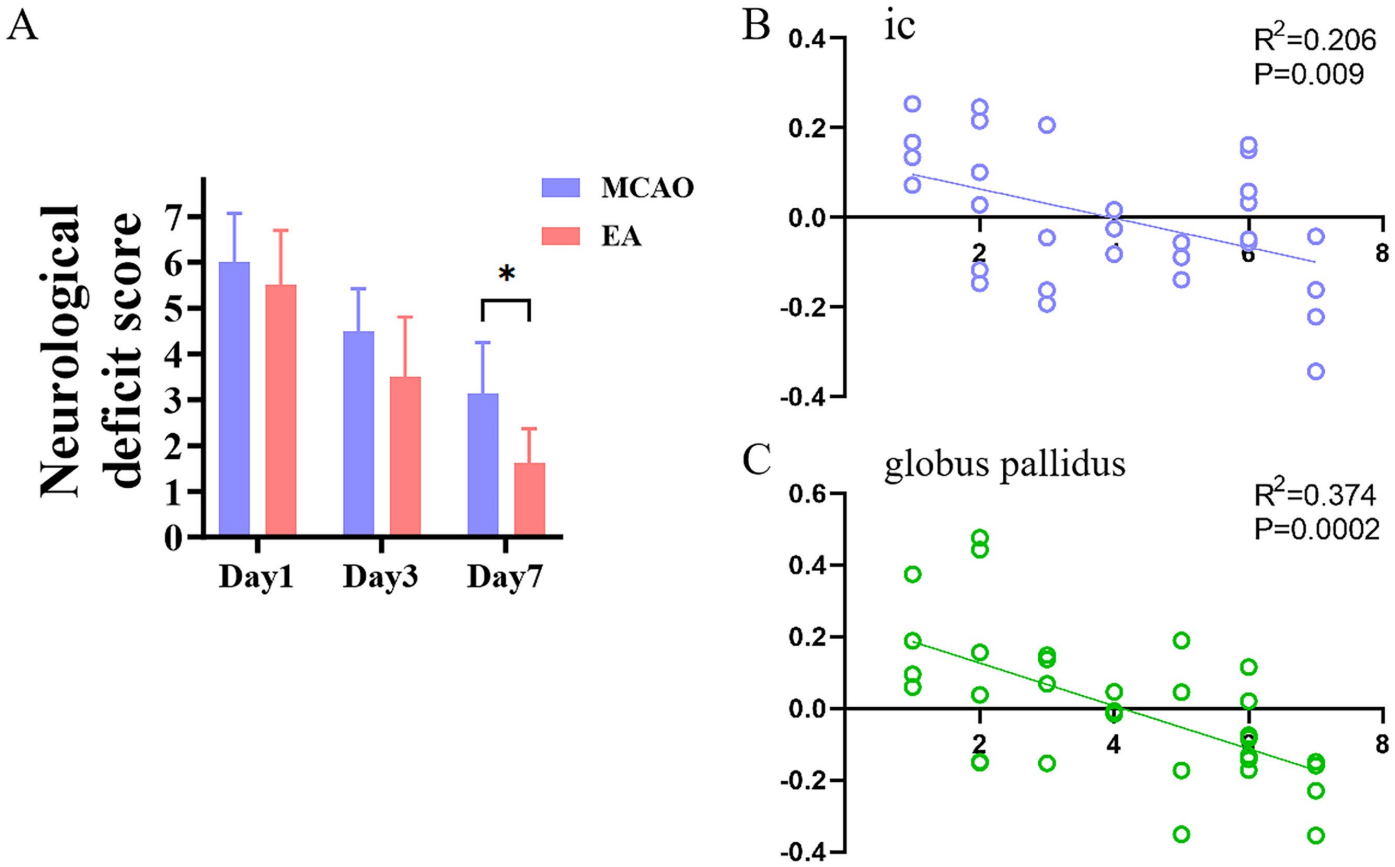
**(A)** The modified neurological severity score of EA and model group at 24 h, Day 3, and Day 7. **(B,C)** Relationships between VMHC of IC and globus pallidus and modified neurological severity score.

As shown in Figure 4A, at 7 days after model, lower Mnss was found in the EA group compared with the Model group (*p* < 0.01). And VMHC values significantly correlated with Mnss in the model and EA group (Figures 4B,C). Significantly negative correlations between the Mnss and VMHC were observed in the ic (*R^2^* = 0.206, *p* = 0.009) and globus pallidus (*R*^2^ = 0.374, *p* = 0.0002).

## 4 Discussion

This study provided sufficient evidence that EA at GV 20 acupoints modulates interhemispheric connectivity in a rat model of ischemic stroke, with specific effects on subcortical motor networks. The results confirmed three important findings: (1) EA selectively enhanced VMHC in basal ganglia-thalamocortical circuits, suggesting that cortical sensory processing may normalize, which may reflect a reduction in compensatory hyperactivation or a rebalance of interhemispheral inhibition. (2) The model of stroke rats without intervention exhibited progressive VMHC decoupling in memory-related and sensorimotor regions; (3) The ic and globus pallidus emerged as primary neuromodulatory targets of EA. These results align with and extend clinical observations.

### 4.1 The key effects of EA on the subcortical motor integration center

The significant increased VMHC in the ic and globus pallidus following EA (Table 2) reveals their role as critical nodes in post-stroke recovery. The ic carries fibers traveling both to and from the cerebral cortex. A significant portion of these fibers constitute the corticospinal tract, which transmits motor signals from the primary motor cortex (23). While the globus pallidus regulates movement inhibition via GABAergic projections to the thalamus (24). Our finding that EA enhanced bilateral synchrony in these regions corroborates human studies where acupuncture improved fractional anisotropy in the internal capsule correlated with the upper extremity Fugl-Meyer scores (25). Mechanistically, EA may normalize glutamate/GABA balance: low-frequency electrical stimulation increases GABA synthesis in the globus pallidus, while high-frequency EA enhance glutamatergic cross-hemispheric signaling via callosal projections (26, 27). This frequency-specific dual modulation (2/15 Hz in this study) may explain the synergistic enhancement of VMHC in the connected striatal-thalamic circuit. It is worth noting that, as a key region for sensory-motor integration, the caudate-putamen showed a significant increase in VMHC after EA. Rodent studies have shown that EA induces higher glucose metabolism levels in this region, thereby mediating motor function recovery (28). Our data suggest EA may facilitate interhemispheric striatal coordination, potentially enabling compensatory movement planning in the unaffected hemisphere (29).

### 4.2 EA-mediated normalization of cortical sensory processing

The observed reduction in VMHC within the somatosensory cortex following EA intervention presented a compelling contrasts to its enhanced activity in subcortical. Post-stroke, the lesioned hemisphere often exhibits decreased activity, while the contralesional hemisphere, particularly homologous sensorimotor areas like somatosensory cortex, may display compensatory hyperactivation (30, 31). Although this hyperactivation is initially adaptive, sustained hyperactivation may reflect impaired processing efficiency and hinder true functional recovery by promoting abnormal interhemispheric inhibition (32). Our finding of decreased somatosensory cortex VMHC after EA contrasted with human stroke studies where acupuncture typically enhances cortical connectivity, suggesting species-specific compensatory mechanisms (33). In rats, somatosensory cortex hyperconnectivity post-stroke may reflect maladaptive reorganization. EA inhibits the ability of the somatosensory cortex to form excessive homologous connections, suggesting targeted normalization of cortical sensory processing. Mechanistically, this may involve EA-induced modulation of inhibitory GABAergic interneurons within the somatosensory cortex (34). This dual effect of enhancing subcortical VMHC while inhibiting excessive cortical VMHC highlights the role of EA in restoring the dynamics of hierarchical networks in the sensorimotor system.

### 4.3 Progressive degeneration of interhemispheric connections after untreated stroke

Our longitudinal analysis of the model group provided critical evidence for the natural course of untreated ischemic injury on interhemispheric connectivity. The significant VMHC reductions observed in the posterodorsal hippocampus, somatosensory cortex, and medial hypothalamus between pre- and post-intervention timepoints revealed a pattern of progressive degeneration decoupling extending beyond the lesion regions. This finding highlighted the dynamic evolutionary nature of interhemispheric connectivity dysfunction. Although the hippocampus is not a primary motor structure, it plays a key role in spatial navigation, contextual memory, and learning processes, which processes that are critical for the reacquisition of motor skills during rehabilitation (35, 36). Stroke-induced hippocampal dysfunction is often associated with involvement of distant brain regions., leading to cognitive impairment and poor rehabilitation outcomes (37). The progressive reduction in the connection strength of the bilateral hippocampus indicated a ongoing disruption in the integration of spatial and contextual information across hemispheres, which may hinder the encoding of new motor memories necessary for rehabilitation. Additionally, as a key hub for coordinator of autonomic, neuroendocrine, and motivational behavior, changes in the VMHC of the medial hypothalamus indicated that stroke has broader systemic effects (38, 39). Weakened ipsilateral hypothalamic connections may be associated with common post-stroke complications such as sleep disorders, fatigue, depression, and autonomic dysfunction, which significantly impact rehabilitation engagement and quality of life (40, 41). These above regions with changed VMHC suggested that therapies aimed at achieving comprehensive recovery must target not only motor circuits but also cognitive, sensory, and autonomic networks that support motor learning and function.

There were several limitation of this study should be clarified. First, while the mNSS provides a valuable global assessment of neurological deficits, the study lacked more specific motor behavioral tests, such as foot-fault test and adhesive removal test. Future study would benefit from a multi-faceted behavioral battery to more precisely correlate brain activity changes with distinct aspects of motor recovery. Secondly, rs-fMRI data were acquired under a combination of medetomidine and isoflurane anesthesia. This regimen was selected as it is a well-established and validated method in rodent fMRI that provides stable anesthesia while better preserving neurovascular coupling and spontaneous neural oscillations compared to alternatives 1–2. Although all groups were subjected to the same protocol, ensuring the validity of group comparisons, the absolute levels of functional connectivity we report are inherent to this anesthetized state. This is a standard consideration in preclinical fMRI, and our findings should be interpreted within this context. Furthermore, the study lacked histological validation, which limits our ability to directly correlate the observed functional connectivity changes with the extent of structural damage. Integrating multimodal imaging and histology in future work will be essential.

## 5 Conclusion

This study confirmed that EA modulates post-stroke recovery through frequency-specific, bidirectional regulation of interhemispheric connectivity. By enhancing subcortical motor integration (basal ganglia-thalamocortical circuits) while suppressing maladaptive cortical reorganization, EA promotes neural network rebalancing. The prevention of hippocampal-hypothalamic decoupling further supports its neuro-protective effects. These mechanistic insights bridge rodent and human studies, providing a foundation for precision EA protocols in stroke rehabilitation. Future work should explore real-time VMHC dynamics during EA stimulation and long-term network remodeling.

## Data Availability

The raw data supporting the conclusions of this article will be made available by the authors, without undue reservation.
